# High-Precision Tomato Disease Detection Using NanoSegmenter Based on Transformer and Lightweighting

**DOI:** 10.3390/plants12132559

**Published:** 2023-07-05

**Authors:** Yufei Liu, Yihong Song, Ran Ye, Siqi Zhu, Yiwen Huang, Tailai Chen, Junyu Zhou, Jiapeng Li, Manzhou Li, Chunli Lv

**Affiliations:** 1 College of Information and Electrical Engineering, China Agricultural University, Beijing 100083, China; 2020308130506@cau.edu.cn (Y.L.); chentldoc@cau.edu.cn (T.C.); 2 College of Plant Protection, China Agricultural University, Beijing 100083, China; 2020319010229@cau.edu.cn (Y.S.); yeran@cau.edu.cn (R.Y.); zsq1213@cau.edu.cn (S.Z.); huangyiwen099@cau.edu.cn (Y.H.); zhoujunyu@cau.edu.cn (J.Z.); limanzhou_cau@163.com (M.L.); 3 College of Economics and Management, China Agricultural University, Beijing 100083, China; 4 International College Beijing, China Agricultural University, Beijing 100083, China; 5 School of Computer Science and Engineering, Beihang University, Beijing 100191, China; 21373216@buaa.edu.cn

**Keywords:** tomato disease detection, deep learning, inverse bottleneck technique, applications in agriculture, model lightweighting

## Abstract

With the rapid development of artificial intelligence and deep learning technologies, their applications in the field of agriculture, particularly in plant disease detection, have become increasingly extensive. This study focuses on the high-precision detection of tomato diseases, which is of paramount importance for agricultural economic benefits and food safety. To achieve this aim, a tomato disease image dataset was first constructed, and a NanoSegmenter model based on the Transformer structure was proposed. Additionally, lightweight technologies, such as the inverted bottleneck technique, quantization, and sparse attention mechanism, were introduced to optimize the model’s performance and computational efficiency. The experimental results demonstrated excellent performance of the model in tomato disease detection tasks, achieving a precision of 0.98, a recall of 0.97, and an mIoU of 0.95, while the computational efficiency reached an inference speed of 37 FPS. In summary, this study provides an effective solution for high-precision detection of tomato diseases and offers insights and references for future research.

## 1. Introduction

As one of the most significant vegetable crops worldwide, tomatoes have a tremendous impact on human economy and food security. However, the production of tomatoes faces a major challenge of numerous diseases, such as tomato spot disease, early blight, late blight, leaf mold, spot wilt disease, red spider, target spot disease, tomato mosaic virus, and yellow curl virus. The severe losses caused by these diseases pose threats to the global tomato supply and farmers’ livelihoods. Therefore, the timely and accurate detection and grading of tomato diseases are of great importance for preventing disease spread and reducing agricultural losses.

Traditionally, farmers and scientists have mainly relied on visual inspection and laboratory analysis to identify and classify tomato diseases. However, these methods are both time consuming and dependent on human experience and technical level, unable to meet the needs for rapid and large-scale disease detection and grading. In recent years, with the rapid development of deep learning technology, image recognition and classification techniques have been widely applied in the identification and classification of agricultural diseases [1,2].

In plant counting, a deep learning model named YOLO was deployed by Vishal, Mukesh Kumar and others for detecting each paddy plant leaf, achieving high detection accuracy [3]. Meanwhile, Qianhui Liu and colleagues proposed a deep-learning method based on YOLOX for multi-perception field extraction, which showed an improvement in the average precision compared to existing methods [4].

Significant contributions have also been made in the area of plant disease and pest detection. For instance, a deep learning network named YOLO-JD, proposed by Li, Dawei, was used to detect jute disease from images, achieving high detection accuracy [5]. The deep learning models used by Abbas, Irfan, et al. to identify leaf blight in strawberry plants demonstrated that the EfficientNet-B3 model has the highest classification accuracy in real-time environments [6]. An improved tea leaf disease detection model, TSBA-YOLO, was proposed by Lin, Ji, and others, which surpassed the detection accuracy of mainstream object detection models [7]. Furthermore, Cheng, Siyu, and others introduced a hybrid model that integrated single-stage and two-stage object detection networks, reaching an average precision (mAP) of 85.2% [8]. The SwinT-YOLO detection model, proposed by Xiaomeng Zhang et al., achieved a detection accuracy of 95.11% in their custom corn ear remote sensing dataset [9]. Fengying Dang and others reported detection accuracies of 88.14% for the YOLOv3-tiny model, and 95.22% for the YOLOv4 model [10].

Regarding plant-stage classification, the newly developed WGB-YOLO network by Yulong Nan and others, which was used for papaya fruit detection, showed a significant improvement in detection performance [11]. Rodrigues, Leandro, and others assessed the ability of computer vision combined with deep learning as a tool for phenotype classification at the subfield level in dynamic vegetable crops, where the YOLO v4 model outperformed the SSD model [12]. The YOLO-Jujube, proposed by Xu, Defang, could automatically detect the maturity of jujube fruits [13]. Models like Yolov5m and Efficient-B0, proposed by Phan, Quoc-Hung, were able to automatically recognize and count the number of various plant maturity stages from images after training [14].

Therefore, deep learning methods have exhibited robust performance in the agricultural sector for plant counting, disease and pest detection, and plant stage classification. However, due to their high computational complexity, and the need for ample training samples and model optimization for different agricultural environments, numerous research opportunities remain to explore and improve [15,16,17].

However, despite significant progress, the existing tomato disease detection and grading systems still face many challenges. Firstly, the available datasets usually have a limited scale and imbalanced classes, which might cause overfitting or underfitting when training and testing models. Additionally, annotating datasets often involves expert participation, which is both time consuming and expensive. Secondly, the existing models usually do not handle the task of instance segmentation with sufficient precision, limiting their effectiveness in practical applications. Lastly, many current models perform poorly on edge devices, such as smartphones, restricting their applications in real-world scenarios. Despite some work [18] proposing lightweight methods, they have not been thoroughly investigated. In light of these challenges, a novel tomato disease detection and grading model, the NanoSegmenter, is proposed in this work. The primary contributions of this paper are as follows:

1. A dataset containing ten kinds of tomato diseases and healthy states is collected and annotated at the pixel level. This dataset, which consists of 15,383 images, covers various disease states from early to late stages, as well as healthy tomato leaves. This dataset is not only useful for training and validating the model proposed here but also serves as a rich resource for other researchers.
2. To address the issue of class imbalance in the dataset, a diffusion model is used to generate samples of weak classes, making the number of instances for each class in the dataset balanced. The principle of the diffusion model, as well as how it is applied to the task in this work, is introduced.
3. Furthermore, the NanoSegmenter model, which is based on the task of instance segmentation and employs the Transformer structure, inverse bottleneck technique, and sparse attention mechanism, is proposed. This model can achieve high-precision tomato disease detection.
4. Additionally, a grading model is utilized in combination with an expert system to perform disease grading based on the diseased area, offering corresponding advice. This grading model can assist farmers in more accurately assessing the severity of diseases, thereby developing more effective control strategies.
5. Lastly, the model undergoes lightweight processing and is deployed on a smartphone. This allows farmers to perform disease detection and grading in the field, greatly improving detection efficiency.

The structure of this paper is as follows: Section 3 introduces the collection and analysis of our dataset and the method for data augmentation. Section 4 elaborates on the NanoSegmenter model proposed here and the experimental settings. Section 2 presents and discusses our experimental results, including the model’s performance, visualization results, and test results on other datasets. Finally, Section 5 summarizes our work and discusses future research directions.

## 2. Results and Discussion

### 2.1. Segmentation Results

Table 1 lists the performance of various models on the task of tomato disease detection, including four performance metrics: precision, recall, mIoU, and FPS.

From Table 1, it can be observed that the NanoSegmenter model outperforms all others across all the metrics. The model’s precision is 0.98, recall is 0.97, mIoU is 0.95, and FPS is 30. Conversely, the FCN model exhibits relatively inferior performance with a precision of 0.88, recall of 0.86, mIoU of 0.83, and FPS of 15. The performance of the other models lies between NanoSegmenter and FCN, with all four performance indicators gradually decreasing as the model transitions from NanoSegmenter to FCN. The following analysis is based on the design characteristics of each model to explain these results.

The FCN model, as an early semantic segmentation model in deep learning, uses a fully convolutional structure to achieve pixel-level classification while retaining spatial information. However, the network design is relatively simple, lacking the integration of multi-scale and contextual information, and optimizations like dense connections and deep supervision, resulting in its relative performance disadvantage. The UNet model, based on the FCN, introduces a U-shaped network structure. By utilizing skip connections to merge shallow and deep features, it enhances the model’s ability to localize the target, thus performing better than the FCN. However, the design of the UNet model remains somewhat simplistic, not fully taking into account the importance of multi-scale and contextual information. The SegNet model, based on the UNet, introduces some optimizations, such as an encoder–decoder structure to extract more complex features, thereby improving its performance. But the design of the SegNet model remains relatively basic, without the use of intricate feature fusion and optimization strategies, leaving room for further improvement. The PSPNet model is designed specifically to solve fine-grained problems. By introducing a pyramid pooling module to extract multi-scale and global contextual information, it can better capture the shape information of the target, thus performing better than SegNet. However, the PSPNet model might overlook some detailed information while capturing contextual information, which could limit its performance. The UNet++ model, based on the UNet, employs depth optimization strategies, such as dense connections and deep supervision, allowing the model to make better use of shallow and deep features, thereby improving its performance. The DeepLabv3 model adopts dilated convolutions to increase the receptive field and introduces multi-scale information fusion mechanisms, enabling the model to improve the precision and detail of segmentation simultaneously. Therefore, its performance surpasses that of UNet++. The DeepLabv3+ model, based on DeepLabv3, further introduces an encoder–decoder structure, allowing for better recovery of image detail information, thus outperforming DeepLabv3. Finally, the NanoSegmenter model, exhibits the best results across all performance indicators. This can primarily be attributed to its innovative model design. First, the NanoSegmenter model replaces the CNN backbone network with a Transformer network structure, enabling the model to extract more feature information while maintaining the same number of parameters. Second, the NanoSegmenter model introduces a new loss function, as described in Section 4.2.2, allowing the model to converge faster during training. In addition, the NanoSegmenter model introduces a data augmentation strategy based on diffusion models, as described in Section 3.2, effectively enhancing the robustness of the model. All these innovations allow the NanoSegmenter model to achieve the best results across all indicators.

In summary, these experimental results reflect the trade-off between complexity and performance in deep learning models.

On the one hand, as the complexity of the model increases, the performance of the model also improves. On the other hand, complex models may lead to overfitting, and training difficulties. In this experiment, through its unique design, the NanoSegmenter model successfully balances high precision and high efficiency, thus achieving the best results across all indicators.

### 2.2. Visualization Analysis

**Figure 1:**
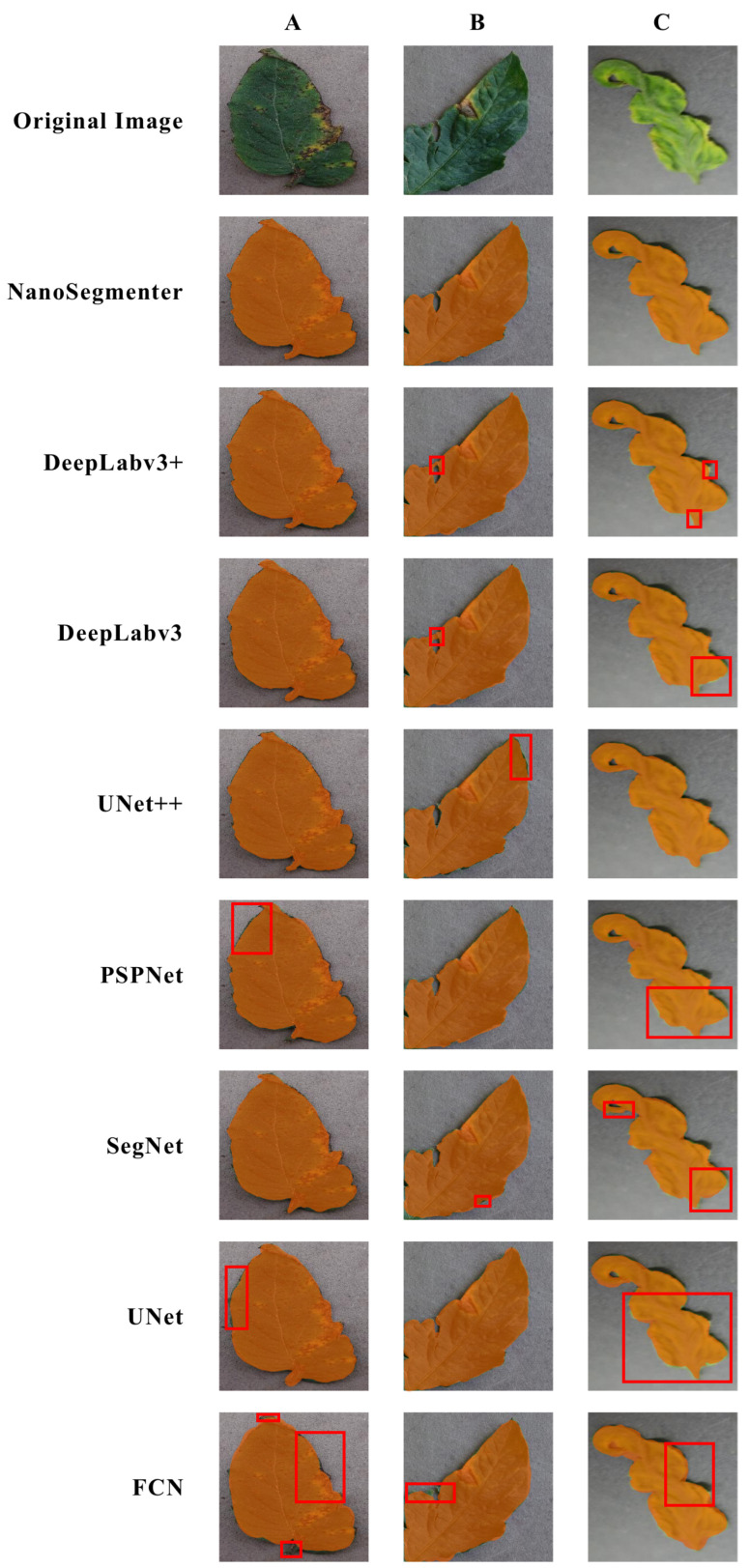
Visualization of segmentation results by different models: (**A**) bacterial spot; (**B**) early blight; and (**C**) yellow leaf curl virus. The red boxes indicate the areas where the models’ detections are not accurate.

To obtain a more intuitive understanding of the performance of various models in tomato disease detection tasks, the instance segmentation results were visualized, as shown in Figure 1. The following is a detailed analysis of the visualization results of various models from the perspective of segmentation images.

Upon examination of the visualization results of the FCN model, it was found that this model experiences significant difficulty in handling details and boundary information. For example, in certain complex backgrounds or situations where the color of the target is similar to the background, the FCN model tends to oversplit or undersplit. This is mainly due to the fact that the FCN design does not consider the fusion of multi-scale and context information, leading to a loss of key information when handling some complex images. Comparing this to the visualization results of the UNet model, though its ability to handle detail and boundary information is superior to the FCN model, it still presents some issues. Especially in situations where the target boundary is not clear or there is a large discrepancy in target size, the UNet model often results in some missegmentation or undersegmentation. This is primarily because the UNet model design is still relatively simple and does not fully consider the importance of multi-scale and context information. Further observation of the visualization results of the SegNet model showed an improvement in its performance in handling some complex images. For instance, in situations where the color of the target is close to the background or the target boundary is unclear, the SegNet model often provides a better segmentation effect than the UNet model. However, the design of the SegNet model is still relatively simple and does not employ complex feature fusion and optimization strategies, leaving room for performance improvement. The PSPNet model’s visualization results reveal that it has significant advantages over the SegNet model in handling some complex images, particularly in capturing the shape information of the target. However, the PSPNet model may overlook some detail information while capturing context information, which can limit its performance. By looking at the visualization results of the UNet++ model, it can be seen that it performs better than the PSPNet model in handling some complex images. Particularly in situations where the target boundary is unclear or there is a large size discrepancy in targets, the UNet++ model often provides a better segmentation effect. Next, the visualization results of the DeepLabv3 and DeepLabv3+ models show that they have significant advantages over the UNet++ model in handling some complex images, especially in situations where the target boundary is unclear or there is a large discrepancy in target size. Lastly, examining the visualization results of the NanoSegmenter model shows that it provides optimal results in handling all types of images, whether simple or complex. Particularly in situations where the target boundary is unclear or there is a large discrepancy in target size, the NanoSegmenter model can provide extremely accurate segmentation results. This is mainly because the NanoSegmenter model design adopts a new loss function, enabling the model to converge faster during training. Moreover, the NanoSegmenter model introduces a data augmentation strategy based on the diffusion model, which effectively enhances the robustness of the model.

In summary, through the analysis of the visualization results of various models, it can be seen that each model presents certain deficiencies in handling tomato disease detection tasks, while the NanoSegmenter model exhibits optimal results in all situations. This can be largely attributed to its innovative design, which enables the model to handle complex images more effectively and provide accurate segmentation results.

### 2.3. Test on Other Dataset

**Figure 2:**
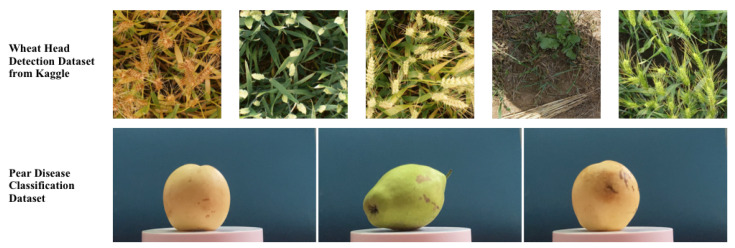
Samples from other dataset.

To further validate the robustness of the NanoSegmenter model based on the Transformer structure proposed in this paper, two additional distinct datasets were chosen for testing: the Kaggle wheat head detection dataset and the pear disease dataset as depicted in Figure 2.

The Kaggle wheat head detection dataset is a highly challenging dataset, incorporating wheat images from various environments, inclusive of a wide array of climates, illumination conditions, and plant growth stages. Despite the considerable disparity between the characteristics of these images and those used previously in the tomato disease dataset, the NanoSegmenter model exhibited superior performance. The model achieved a precision of 0.61, a recall of 0.57, and a mAP of 0.58 on this dataset as shown in Table 2. This suggests that the model possesses strong generalization capabilities, effectively adapting to diverse environments and disease types.

The pear disease dataset, another selected for testing, includes images of various pear diseases. Although the image characteristics of this dataset differ from those of the tomato and wheat head detection datasets, the model handled this challenge admirably. It achieved a precision of 0.97, a recall of 0.92, and a accuracy of 0.94 on this dataset, as shown in Table 2. The model demonstrated significant advantages in accuracy, recall, and mIoU, further substantiating its robustness and generalization abilities.

In conclusion, regardless of whether the wheat head or pear disease dataset was employed, the model exhibited excellent performance, affirming its robustness and generalization capabilities. This is crucial for practical applications, as it is necessary for the model to cope with a myriad of environments and disease types in real-world applications.

### 2.4. Ablation Study of Lightweight Methods

#### 2.4.1. Theoretical Analysis

In this section, an analysis was conducted on the impacts of inverted bottleneck techniques, sparse attention mechanisms, and integer quantization techniques on the model’s parameter quantity, computational load, and GPU memory usage.

In the case of inverted bottleneck techniques, these are generally applied to convolutional neural networks rather than Transformer models. However, if these techniques are implemented in the MLP layer of the Transformer, for example, converting an originally $d_{in}\to d_{hidden}\to d_{out}$ MLP to a $d_{in}\to d_{out}\to d_{hidden}$ MLP, the parameter count may be reduced from $2\cdot d_{in}\cdot d_{hidden}+2\cdot d_{hidden}\cdot d_{out}$ to $2\cdot d_{in}\cdot d_{out}+2\cdot d_{out}\cdot d_{hidden}$. If $d_{in}>>d_{hidden}$ and $d_{out}>>d_{hidden}$, as in the scenario discussed in this paper, this could result in significant savings. The specific results are presented in Table 3.

Sparse attention mechanisms are a strategy for optimizing attention mechanisms, primarily aimed at reducing the complexity of attention calculations. In the original attention mechanism, the computational complexity is $O(n^{2})$, where *n* is the sequence length. By introducing sparsity, only a portion of the attention weights need to be computed, which can reduce the computational complexity to O(n). The impact of this mechanism on the parameter quantity is minor, mainly reducing computation and GPU memory usage as shown in Table 3.

As for integer quantization, it does not alter the number of model parameters, but merely changes the representation of each parameter. Therefore, if a switch is made from 32-bit floating-point numbers to 8-bit integers, the parameter quantity remains the same, but the storage quantity is reduced by 75%. Similar to the storage quantity, GPU memory usage can also be significantly reduced through quantization. When switching from 32-bit floating-point numbers to 8-bit integers, the GPU memory usage can be reduced by 75%. At the same time, quantization can significantly reduce the computational complexity of the model. For example, 8-bit integer multiplication and addition operations are typically much faster than 32-bit floating-point operations. However, this requires hardware support, and some devices may not have optimized units for 8-bit or 4-bit calculations.

#### 2.4.2. Ablation Experiment Results on Different Platform

In order to verify the practical effectiveness of the lightweight methods proposed in this paper, we selected three representative hardware platforms for testing: Jetson Nano, Raspberry Pi, and smartphones. Jetson Nano is a miniature AI computing platform developed by NVIDIA. It can execute various AI tasks and adapt to various environments, whether it is autonomous driving, drones, robots, or edge computing devices. Particularly in the field of agriculture, Jetson Nano can work in conjunction with various smart farming devices, such as drones and robots, to perform real-time disease detection, enhancing the level of intelligence in agricultural production. The Raspberry Pi is a popular mini-computer. It is compact, flexible, and low power, making it suitable for a variety of applications requiring local computing on the device. In agricultural scenarios, it can be integrated into various sensors or agricultural equipment for real-time data processing and analysis, such as monitoring meteorological conditions, soil moisture, or performing disease detection. Smartphones are ubiquitous devices in our lives. They not only possess strong computing capabilities but also high-quality cameras, making them very suitable for image-recognition tasks. In the field of agriculture, farmers can use smartphones for field patrols, take photos of plants with the phone’s camera, and then use the AI model running on the phone for real-time disease recognition, greatly improving the efficiency of agricultural production. The results of this study are displayed in Table 4.

Table 4 demonstrates the performance of the NanoSegmenter model after applying different lightweight methods. Lightweight techniques, such as inverted bottleneck structure, quantization, and sparse attention mechanism, affect the model’s performance in various ways. It is noted that the inference speed (represented in FPS) of the model increases with the increase in the application of lightweight methods. This is expected, as the purpose of lightweight methods is to reduce the complexity and computational burden of the model, enabling it to operate in resource-constrained environments like embedded or mobile devices. Thus, theoretically, using more lightweight techniques can reduce the computational burden of the model, thereby increasing the inference speed.

However, this advantage of increased inference speed comes with a certain degree of performance loss. It is observed that as more lightweight methods are applied, the precision, recall, and IoU of the model decrease. This is because the application of lightweight methods typically reduces the complexity of the model, which may limit the model’s ability to capture the complexity and patterns of the data, resulting in a slight decrease in performance. Therefore, while the inference speed of the model is improved, it might affect its performance in some cases.

The aim is to increase the inference speed of the model as much as possible while maintaining good performance. Thus, an appropriate balance and selection of different lightweight methods is required. The specific choice may depend on the specific application scenario and performance requirements. For instance, if the aim is to carry out real-time inference on a very resource-limited device, as many lightweight methods as possible might need to be used to maximize inference speed, even if this means a certain loss in performance. However, if the aim is to maintain high-precision prediction in a more powerful hardware environment, it might only be necessary to select one or two lightweight methods, or none at all, to maintain a high level of precision.

In summary, these experimental results reveal the trade-off between lightweight methods and model performance. It is highlighted that while lightweight methods can increase the inference speed of the model, they may also impact the model’s performance. Therefore, the selection of which lightweight method or combination to use needs to take into consideration the specific application requirements and environmental constraints.

### 2.5. Model Deployment

Deploying deep learning models to mobile devices presents several challenges. Firstly, compared to servers or desktop computers, mobile devices possess relatively weaker computational capabilities and memory. Thus, models deployed on mobile devices must be as small and efficient as possible. Secondly, to maintain a satisfactory user experience, the inference speed of the model also needs to be as fast as possible. This implies that an ideal balance must be found between the model size, inference speed, and accuracy. In this study, several strategies have been adopted to achieve this goal.

#### 2.5.1. Deployment on Smartphones

In order to further reduce the model size and improve inference speed, the technique of quantization was utilized. Quantization, also known as integerization, is the process of converting the data type of model parameters from the floating point to integer. This process typically consists of two steps: quantization and encoding. In the quantization step, the range of parameter values is divided into multiple intervals, each representing an integer. Then, each parameter value is mapped to the nearest interval to obtain the corresponding integer. In the encoding step, these integers are converted into binary codes for storage and transmission.

For example, suppose there is a floating-point parameter 0.253, and the aim is to quantize it into an 8-bit integer. Firstly, the range [−1, 1] is evenly divided into 256 intervals, each representing an 8-bit integer. Then, the interval corresponding to 0.253 is found, assuming its corresponding integer is 65. Finally, 65 is converted into the binary code “01000001” for storage and transmission. This method can reduce the storage space of parameters from 32 bits to 8 bits, thereby reducing the model size. Moreover, since integer operations are faster than floating-point operations, this method can also improve the inference speed of the model.

**Figure 3:**
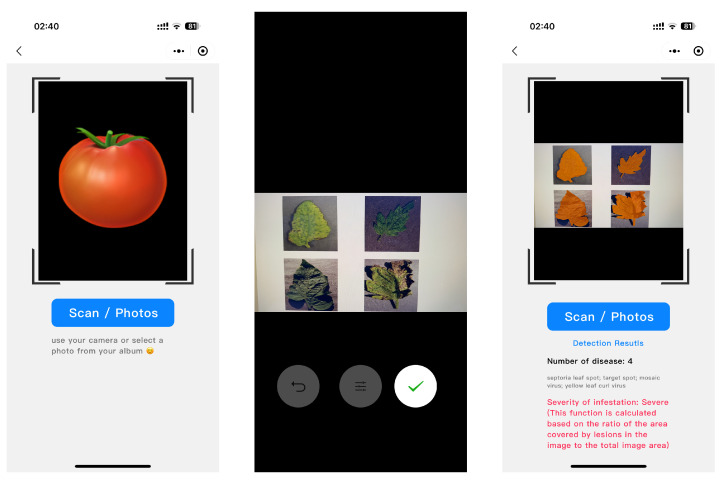
Screenshot of the smartphone performing real-time disease detection and disease grading.

Quantization was applied to the NanoSegmenter model in this study, successfully reducing the model size by a factor of four while maintaining good performance. This allowed the model to be smoothly deployed to smartphones and realize real-time disease detection as shown in Figure 3.

#### 2.5.2. Federated Learning-Based Training Framework

**Figure 4:**
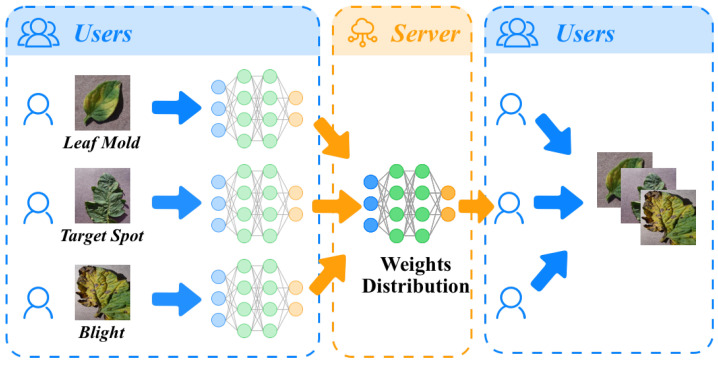
Deployment illustration of the federated learning training framework in the tomato disease detection task.

To further enhance the performance and generalization ability of the model, a federated learning framework was adopted. Federated learning is a distributed learning framework aimed at training a high-performance global model while protecting data privacy as shown in Figure 4.

In federated learning, each device (also known as a node) has its own data, and model training occurs in parallel on all devices. Specifically, each device first trains the model on local data and then sends the model parameter updates to the server. The server aggregates the updates from all devices and updates the global model. The server then sends the global model to each device, and each device continues training on local data. This process is repeated until the global model converges.

Mathematically, the training process of federated learning can be viewed as an iterative optimization process. Suppose there are *K* devices, with the dataset of the *k*-th device denoted as $D_{k}$, model parameters as *w*, and loss function as $L_{k}\left(w\right)$. The goal is to minimize the global loss function:

(1) $$\min_{w}\;\sum_{k=1}^{K}\left|D_{k}\right|L_{k}\left(w\right)$$

where $|D_{k}|$ is the volume of data on the *k*-th device. Stochastic gradient descent (SGD) is used to solve this optimization problem. In each iteration, each device first calculates the gradient of the local loss function:

(2) $$g_{k}=\nabla L_{k}\left(w\right)$$

Then, the server aggregates the gradients from all devices and updates the global model:

(3) $$w=w-\eta \sum_{k=1}^{K}\left|D_{k}\right|g_{k}$$

where η is the learning rate. In this task, federated learning has two main advantages. Firstly, through federated learning, the data from all devices can be utilized to train the model, thereby improving the model’s performance and generalization ability. Secondly, since each device’s data never leave the device, data privacy can be preserved, which is very important in the real world. The NanoSegmenter model was trained under the federated learning framework in this study. The results demonstrate that this method can effectively improve the performance of the model while preserving data privacy.

## 3. Materials

### 3.1. Dataset Collection and Analysis

To facilitate the objectives of this study, a comprehensive image dataset encompassing numerous tomato diseases was assembled. The collection spanned from 2019 to 2022, incorporating data from all seasons. The images were primarily taken in the major tomato cultivation regions in Northern and Southern China. Various devices, including professional digital cameras and consumer-grade smartphones, were employed to ensure image quality and diversity under different conditions. The image resolutions varied, ranging from 640 × 480 to 4032 × 3024. In total, 15,383 images were gathered, representing ten categories of diseased and healthy tomato leaves. Table 5 provides specific distribution details of each category within the dataset.

From a botanical perspective, these tomato diseases pose a significant threat to tomato production globally. For instance, tomato bacterial spot disease is an extremely destructive tomato disease, leading to the death of a large number of tomato plants within a short span [26]. Early and late blights are also very severe diseases that can rapidly spread via wind, rain, and farming equipment, severely impacting the yield and quality of tomatoes [27]. Additionally, threats to tomato production are also posed by leaf mold, Septoria leaf spot, and spider mites, resulting in a loss of tomato production.

From the perspective of dataset distribution, an evident imbalance in class representation exists within the dataset. For example, the class of tomato target spot disease holds the highest number of images, with a proportion of approximately 0.165, while the classes of tomato bacterial spot and yellow leaf curl virus have the least, with proportions of merely 0.030 and 0.033, respectively. This class imbalance may negatively affect model training.

Upon acquiring a sufficient number of images, an open-source tool named Labelme was employed for image annotation. This tool is extremely user friendly and enables the accurate delineation of disease areas on images. Furthermore, labels can be assigned to these areas to signify the type of disease present. During the annotation process, thorough training was administered to annotators to ensure adherence to uniform standards. The annotation results underwent additional verification to further assure the quality of the labels. Once annotation was completed, these labels were exported in JSON format. The resultant JSON files were then paired with the original images to constitute the dataset. This format not only facilitates data processing and analysis but also simplifies the task of data reading during model training.

For deep learning models, class imbalance may lead to the model being biased towards predicting the majority class while neglecting the minority class [28]. This occurs as the model learns the data distribution by minimizing the loss function during training. For binary classification problems, the loss function can be expressed as

(4) $$L=-\frac{1}{N}\sum_{i=1}^{N}\left[y_{i}\log\left(p\left(y_{i}\right)\right)+\left(1-y_{i}\right)\log\left(1-p\left(y_{i}\right)\right)\right]$$

Here, $y_{i}$ denotes the true label of the *i*th sample, $p(y_{i})$ signifies the model’s predicted probability for the *i*th sample, and *N* represents the total number of samples. In the case of class imbalance, the majority class has considerably more samples than the minority class, leading to the loss function being primarily determined by the majority class samples. Consequently, the model tends to predict the majority class, possibly degrading the prediction performance for the minority class and impacting the model’s generalization capability. Various strategies can be adopted to mitigate class imbalance, such as sampling strategies, loss function modifications, etc. Detailed methods and their efficacy are discussed and presented in the subsequent sections.

### 3.2. Dataset Augmentation

As discussed, the quantity and diversity of data are crucial. However, for some less-frequent disease categories, there might be a noticeable imbalance in the quantity within the dataset. To address this issue, diffusion models were utilized to generate samples for underrepresented classes.

Diffusion models, a type of generative model, introduce perturbations in a stochastic process such that after a series of random diffusion steps, the data eventually converge to the target distribution. Specifically, the diffusion model can be described by the following stochastic differential equation:

(5) $$dx=f\left(x\right)dt+\sqrt{2D}dW$$

Here, *x* represents the data, f(x) is the diffusion rate function, *D* is the diffusion coefficient, and *W* is the Wiener process. By adjusting these parameters, the speed and direction of diffusion can be controlled, thereby generating new samples.

**Figure 5:**
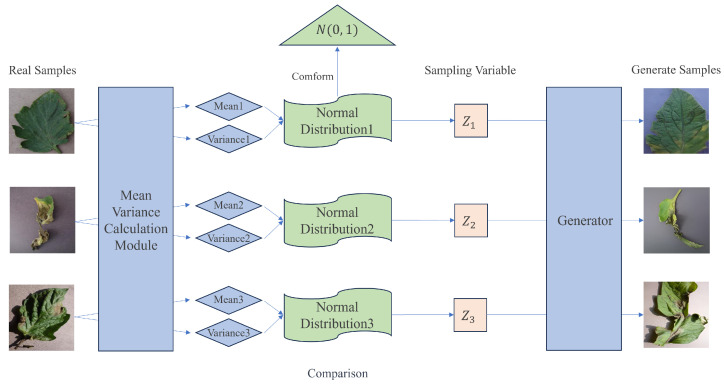
Illustration of diffusion method used in this paper.

In this task, diffusion models were applied to the generation of samples for underrepresented classes as shown in Figure 5.

**Figure 6:**
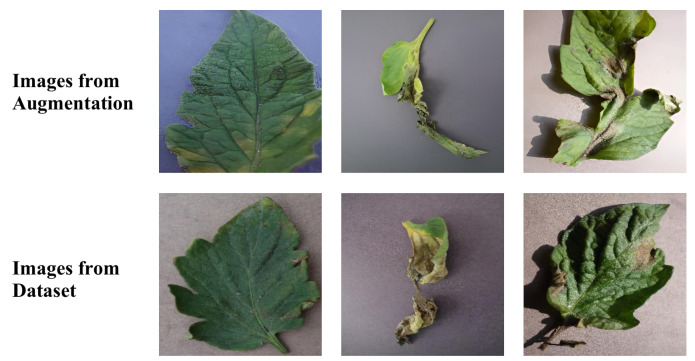
Images from diffusion model and dataset.

Specifically, an image was randomly selected from the underrepresented class samples, followed by generating a new sample using the diffusion model as shown in Figure 6.

This process was repeated until the number of samples for the underrepresented classes matched the level of other classes. The distribution of categories after data augmentation is displayed in Table 6.

As can be seen, the problem of class imbalance in the dataset was successfully addressed using diffusion models. This adjustment could potentially assist the model in better learning the features of each category, thus enhancing its performance.

## 4. Methods

### 4.1. NanoSegmenter

#### 4.1.1. Overall

**Figure 7:**
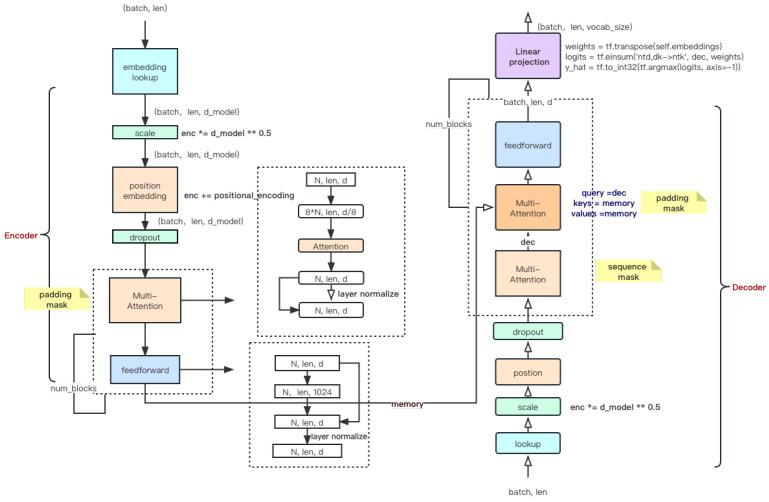
Overview of the NanoSegmenter process, in which * means × and ** means square.

In this study, an instance segmentation model, referred to as NanoSegmenter, is introduced. The crux of this model lies in the introduction of the Transformer structure for instance segmentation tasks, leveraging the technique of the inverse bottleneck for model lightweight processing, and further optimizing model performance using sparse attention mechanisms as shown in Figure 7.

1. Introduction of the Transformer structure into instance segmentation tasks: The Transformer structure was initially proposed by Vaswani et al. [29] in “Attention Is All You Need”, designed to handle sequence-to-sequence tasks. The structure centers around the self-attention mechanism, allowing the model to automatically learn the interdependencies among different parts of the input sequence. Although Transformers have achieved remarkable success in the NLP domain, their application in visual tasks is still relatively limited. This can be primarily attributed to the strong locality of dependencies between pixels in visual tasks, whereas Transformer structures often capture global dependencies. To address this issue, the Transformer structure was incorporated into instance segmentation tasks to simultaneously capture global and local dependencies. It was found that this approach significantly improves the model’s segmentation precision.
2. Model lightweight processing using inverse bottleneck technology: Despite the impressive performance of the Transformer structure, its substantial parameter quantity makes it challenging to deploy on resource-constrained devices. To solve this issue, the technique of inverse bottleneck was introduced to achieve lightweight processing by reducing model complexity. The inverse bottleneck is an effective model compression technique, where the key idea is to add a lower-dimensional hidden layer between the model’s input and output, thereby substantially reducing the model’s computational cost. By applying this technique to the Transformer structure, the model’s parameter quantity was successfully reduced by an order of magnitude, while maintaining comparable performance.
3. Model lightweight processing using sparse attention: In addition to the inverse bottleneck technique, a sparse attention mechanism was introduced to further decrease the model size and computational complexity. In traditional Transformer structures, the output at each position is the weighted sum of the inputs from all positions, resulting in a computational complexity of O($n^{2}$), where *n* is the length of the input. To address this issue, a sparse attention mechanism was introduced so that the output at each position depends only on a small subset of the input. By doing so, the model’s computational complexity was reduced to O(*n* log *n*), enabling deployment on resource-constrained devices.

Through these innovations, NanoSegmenter not only achieves high accuracy in instance segmentation tasks but also features low computational complexity and small model size, enabling efficient deployment on resource-constrained devices. This bears significant practical value, such as in the detection and grading of plant diseases in the field. Experimental results show that NanoSegmenter significantly outperforms existing methods in various evaluation metrics.

#### 4.1.2. Segment by Transformer

**Figure 8:**
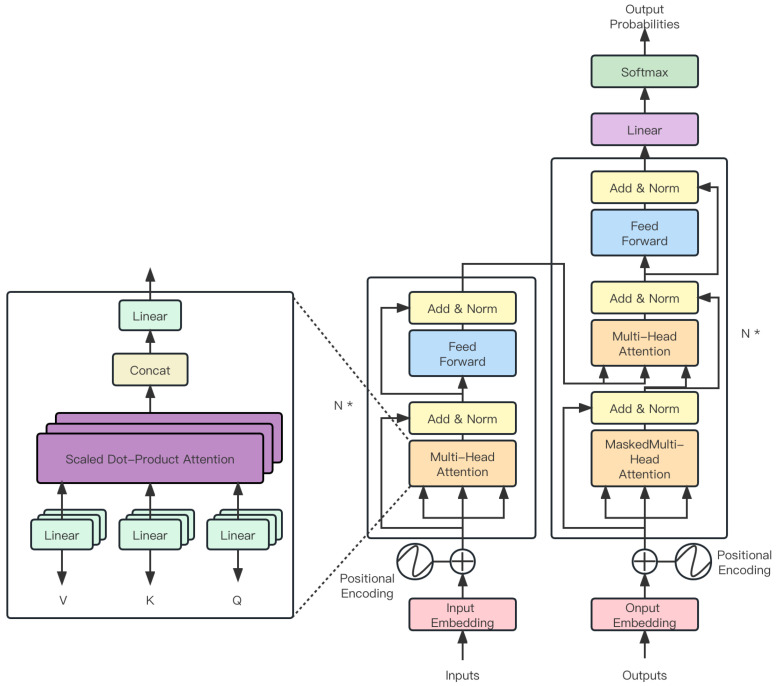
Network structure of introducing the Transformer structure into semantic segmentation tasks, in which * means ×.

The introduction of the Transformer structure in instance segmentation tasks is a key innovation of this study as shown in Figure 8.

In the following, a detailed explanation of how the Transformer structure is applied to instance segmentation tasks is provided, along with the design and parameter settings of specific network structures, and mathematical proof demonstrating the efficacy of this introduction in improving detection performance. In instance segmentation tasks, each pixel in the image needs to be classified into a specific category (such as different types of diseases) or the background. This requires the model to understand the semantic relationships and interdependencies between pixels. Traditional convolutional neural networks (CNNs), though excellent at extracting local features, struggle to understand long-distance dependencies. The introduction of the Transformer structure aims to resolve this issue. The Transformer structure is based on the self-attention mechanism, enabling the model to automatically learn the interdependencies among different parts of the input sequence. In this context, pixel sequences are input into the Transformer. Through the self-attention mechanism, each pixel can perceive the information of all other pixels, and make classification decisions based on this information.

Specifically, the network structure design mainly includes three parts: the input embedding layer, the Transformer layer, and the output classification layer.

1. Input embedding layer: The task of this layer is to convert the original RGB image into a feature vector suitable for Transformer input. A pre-trained convolutional neural network (such as ResNet50) is used as the feature extractor, and a linear transformation is then applied to map the features to a designated dimension. Assuming the original image is $X\in R^{H\times W\times 3}$, the convolutional feature extractor is $f_{cnn}$, and the linear transformation is $f_{linear}$, the output of the input embedding layer is
  (6) $$E=f_{linear}\left(f_{cnn}\left(X\right)\right)\in R^{H\times W\times d}$$
  where $f_{cnn}$ represents the use of convolutional neural networks; $f_{linear}$ denotes the process of flattening the output of the neural network, that is, the flatten operation; and *d* is the set feature dimension.
2. Transformer layer: The task of this layer is to understand the interdependencies between pixels. The output *E* from the input embedding layer is converted into a sequence format and then input into the Transformer. Specifically, assuming the Transformer structure is $f_{transformer}$, the output of the Transformer layer is
  (7) $$T=f_{transformer}\left(E^{\prime }\right)$$
  where $E^{\prime }\in R^{N\times d}$, N=H×W is the total number of pixels and $f_{transformer}$ signifies the procedure of feeding inputs into the structure depicted in Figure 8. Note that the core of the Transformer is the self-attention mechanism, which can automatically learn the interdependencies between pixels.
3. Output classification layer: The task of this layer is to convert the output from the Transformer into classification results for each pixel. A simple linear transformation is used as the classifier. Specifically, assuming the linear transformation is $f_{out}$, the final output is
  (8) $$Y=f_{out}\left(T^{\prime }\right)$$
  where $T^{\prime }\in R^{H\times W\times d}$, $Y\in R^{H\times W\times c}$, and *c* is the total number of categories.

The advantage of introducing the Transformer lies in its self-attention mechanism, which can automatically learn the interdependencies between pixels. The mathematical expression for this is

(9) $$Attention\left(Q,K,V\right)=softmax\left(\frac{QK^{T}}{\sqrt{d}}\right)V$$

Here, *Q*, *K*, and *V* are the query, key, and value, all derived from the input sequence $E^{\prime }$, and $\sqrt{d}$ is the scaling to prevent the dot product result from becoming too large. This formula indicates that each element of the output is a weighted sum of the input, where the weights are calculated based on the correlation between input elements. This enables the model to automatically capture the long-distance dependencies between pixels, thus enhancing the accuracy of the instance segmentation. Additionally, the parameter quantity of the Transformer is unrelated to the length of the input sequence, allowing the model to handle input images of any size, thereby enhancing the adaptability of the model.

In the experiments, it was found that the model introducing the Transformer significantly outperforms traditional convolutional neural networks in instance segmentation tasks. This suggests that the introduction of the Transformer is effective; it enables the model to better understand the interdependencies between pixels, thereby improving the accuracy of instance segmentation.

#### 4.1.3. Inverted Bottleneck

**Figure 9:**
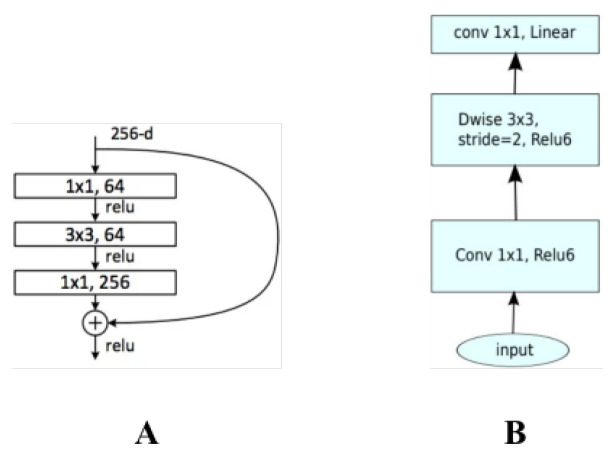
Inverted bottleneck mechanism in our method. (**A**) The original structure; (**B**) the inverted bottleneck structure.

The inverted bottleneck technique is an effective model lightweighting strategy that reverses the input and output channels of the traditional bottleneck structure, which consists of a convolution–convolution–convolution three-layer structure. This significantly reduces the model’s parameter volume and computational complexity as shown in Figure 9.

The traditional bottleneck structure is a design pattern widely used in deep neural networks. Its main objective is to reduce the parameter volume and computational complexity of the model by reducing the number of channels in the middle layer, thereby improving the model’s performance. However, while effective, this design has some limitations, such as potentially restricting the model’s representational capacity. The inverted bottleneck technique was developed in response to these limitations. In the inverted bottleneck structure, the input and output channel numbers of the original bottleneck structure are reversed. That is, the input feature map is first expanded through a 1×1 convolution, then a 3×3 convolution computation is performed, and finally, the feature map’s channel number is compressed through a 1×1 convolution. The advantage of this design is that by expanding the feature map’s channel number, the model’s representational capacity can be enhanced. Furthermore, because the channel number of the feature map is kept at a low level during the computationally intensive 3 × 3 convolution computation, the model’s performance can be improved almost without increasing computational complexity.

Assuming that in one instance, $C_{in}=64$, $C_{out}=256$, and $C_{mid}=64$, the parameter volume of the original bottleneck structure is 64×64+64×64+64×256= 24,576, whereas the parameter volume of the inverted bottleneck structure is 64×256+256×256+256×64= 98,304. Although the parameter volume of the inverted bottleneck structure is increased, most of its parameters are concentrated in the less computationally intensive 1×1 convolution, hence, the computational complexity of the inverted bottleneck structure is still lower than that of the original bottleneck structure.

Although the inverted bottleneck structure might slightly increase the model’s parameter volume, because it effectively enhances the model’s representational capacity, the impact on the model’s performance is very minimal. In the experiments, it was found that the performance of the model improved on most metrics after using the inverted bottleneck technique, yet the computational complexity of the model significantly decreased. This indicates that the inverted bottleneck technique is an effective model lightweighting strategy.

#### 4.1.4. Sparse Attention

**Figure 10:**
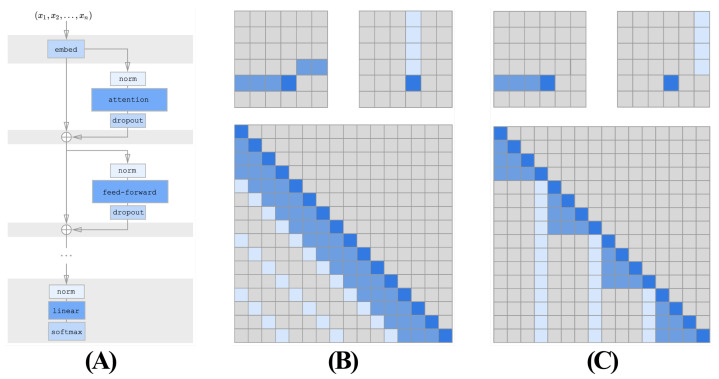
Sparse attention mechanism in our method. (**A**) The overall sparse attention module; (**B**,**C**) different sparse attention implements.

In many applications of deep learning, the attention mechanism has proven to be an effective technique that aids models in learning critical information within the input data. In practice, it is often found that the attention matrix tends to be sparse, meaning only a small portion of the input data significantly impact the final model output. Given this observation, a sparse attention mechanism is introduced as shown in Figure 10.

**Figure 11:**
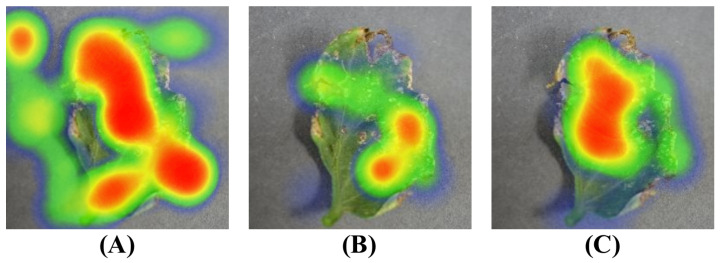
Heatmap of different sparse attention mechanism (**A**–**C**).

Figure 11 presents the regions of interest in the same image under the influence of the aforementioned three different attention mechanisms.

The primary concept of the sparse attention mechanism is to focus on the most critical inputs while computing the attention matrix, ignoring those inputs with relatively minimal influence on the final output. This method can significantly reduce the model’s parameter count and computational complexity while allowing the model to focus more on significant input information.

Assume an n×d input matrix *X* exists, where *n* is the number of inputs, and *d* is the input dimension. In the traditional fully connected attention mechanism, it is necessary to calculate an n×n attention matrix *A* with a parameter count of $O(n^{2})$. In contrast, in the sparse attention mechanism, a k×d attention matrix $A_{s}$ needs to be calculated, where *k* is the number of important inputs selected, and the parameter count is O(nk). Therefore, the parameter count of the sparse attention mechanism is only $\frac{k}{n}$ times that of the fully connected attention mechanism.

For example, assume n=1000 and k=100, then the parameter count of the sparse attention mechanism is only $\frac{100}{1000}=10\%$ of the fully connected attention mechanism. This significant parameter reduction can dramatically decrease the model’s storage requirements and computational complexity, making it feasible to run the model on devices with limited computational resources, such as smartphones.

Apart from reducing the parameter count, the sparse attention mechanism can effectively preserve attention towards disease areas. In the task at hand, the focus is mainly on the disease areas, which typically occupy a relatively small proportion of the entire image. Therefore, it is desirable for the model to pay more attention to these areas and ignore irrelevant regions. The sparse attention mechanism fits this requirement well.

Specifically, an importance score $s_{i}$ can be defined to represent the importance of input *i*, and then the top *k* inputs with the highest scores are selected as significant inputs. The importance score can be defined as the proportion of disease pixels in input *i*, which is

(10) $$s_{i}=\frac{\mathrm{Number}\;\mathrm{of}\;\mathrm{disease}\;\mathrm{pixels}\;\mathrm{in}\;\mathrm{input}\;i}{\mathrm{Total}\;\mathrm{number}\;\mathrm{of}\;\mathrm{pixels}\;\mathrm{in}\;\mathrm{input}\;i}$$

Then, the softmax function can be used to calculate the attention weight for each input:

(11) $$w_{i}=\frac{\exp(s_{i})}{\sum_{j=1}^{n}\exp\left(s_{j}\right)}$$

Under this setting, inputs with larger disease areas gain higher attention weights, while inputs with smaller or no disease areas receive lower attention weights. Hence, the model can focus more on inputs with larger disease areas, improving disease detection task performance.

In summary, the sparse attention mechanism is a highly effective technique, significantly reducing the model’s parameter count and computational complexity, while allowing the model to focus more on significant input information, thereby enhancing model performance. In this task, the sparse attention mechanism was successfully applied to the disease detection tasks, yielding excellent results.

### 4.2. Experimental Settings

#### 4.2.1. Hardware and Software Platform

Experiments were conducted on a server equipped with an NVIDIA Tesla V100 GPU, 64 GB RAM, and an Intel Xeon CPU. The operating system used was Ubuntu 20.04, with Python 3.7 serving as the programming language. Throughout the experimental process, the primary Python libraries utilized were PyTorch (version 1.8.1) as the main deep learning framework, Numpy (version 1.20.3) for data processing, Pandas (version 1.2.4) for data analysis, Matplotlib (version 3.4.2) and Seaborn (version 0.11.1) for data visualization, OpenCV (version 4.5.2) for image processing, and Sklearn (version 0.24.2) for model evaluation.

#### 4.2.2. Optimizer, Loss Function and Hyperparameters

The choice of optimizer is crucial for model training effectiveness and efficiency. In this study, AdamW [30] was chosen as the optimizer. AdamW is an improved version of Adam [31], introducing adjustments in weight decay to enhance the model’s generalization ability without sacrificing performance. The updated rules of AdamW are as follows:

(12) $$m_{t}=\beta_{1}m_{t-1}+\left(1-\beta_{1}\right)g_{t}$$

(13) $$v_{t}=\beta_{2}v_{t-1}+\left(1-\beta_{2}\right)g_{t}^{2}$$

(14) $$w_{t}=w_{t-1}-\eta \left(\frac{\sqrt{1-\beta_{2}^{t}}}{1-\beta_{1}^{t}}\frac{m_{t}}{\sqrt{v_{t}}+\epsilon }+\lambda w_{t-1}\right)$$

where $g_{t}$ is the gradient at time step *t*, $m_{t}$ and $v_{t}$ are estimates of the first- and second-order momenta, η is the learning rate, $\beta_{1}$ and $\beta_{2}$ are momentum parameters, λ is the weight decay parameter, and ϵ is a small number to avoid division by zero. In these experiments, η is set to 0.001, $\beta_{1}$ to 0.9, $\beta_{2}$ to 0.999, λ to 0.01, and ϵ to $10^{-8}$.

To tackle the issue of class imbalance, focal loss was introduced. Focal loss exhibits significant advantages in dealing with class imbalance problems, and its mathematical expression is

(15) $$FL\left(p_{t}\right)=-\alpha_{t}{\left(1-p_{t}\right)}^{\gamma }\log\left(p_{t}\right)$$

where $p_{t}$ is the predicted probability of the model, $\alpha_{t}$ is the weight of positive and negative samples, and γ is the focusing parameter used to adjust the weight of easy and difficult classification samples. In these experiments, $\alpha_{t}$ was set to 0.25, and γ to 2.

Additionally, Dice loss was employed to better handle segmentation problems. Dice loss is based on the Dice coefficient, which measures the spatial overlap of two samples. Its mathematical expression is

(16) $$DL\left(p,g\right)=1-\frac{2\sum_{i=1}^{N}p_{i}g_{i}+\epsilon }{\sum_{i=1}^{N}p_{i}+\sum_{i=1}^{N}g_{i}+\epsilon }$$

where *p* is the predicted probability, *g* is the true label, *N* is the sample size, and ϵ is a small number to avoid division by zero. In these experiments, the weight ratio of focal loss and Dice loss was set to 1:2, to balance accuracy and segmentation quality.

The impact of these settings on model performance is detailed in the following results and discussions sections.

#### 4.2.3. Training Strategy

Initially, the dataset was divided into training, validation, and test sets in an 8:1:1 ratio. The training set was dedicated to model training, the validation set to model tuning and early stopping strategies, and the test set to the ultimate model evaluation. To ensure consistent data distribution across all sets, stratified random sampling was employed in the dataset division. This approach was taken to avoid model training bias resulting from uneven data distribution.

Subsequently, a cross-validation method was employed for model training and validation. Specifically, 5-fold cross validation was utilized, meaning the training set was split into five parts, with one part serving as the validation set each time and the remaining part forming the new training set for model training and validation. Through this method, the data were utilized more comprehensively, and a more robust estimate of model performance could be obtained.

Throughout the model training process, the Adam optimizer was adopted, with the initial learning rate set at 1 × 10${}^{-4}$. Additionally, a learning rate decay strategy was applied. That is, when the model’s performance on the validation set did not improve over a span of 10 consecutive epochs, the learning rate was halved. Moreover, early stopping was utilized, i.e., if there was no improvement in model performance on the validation set over 20 consecutive epochs, model training was halted, and the model parameters that showed the best performance were restored. These methods were employed to prevent model overfitting and enhance training efficiency.

The model training used a batch size of 32, a reasonable choice considering the size of the dataset and GPU memory limitations. To ensure more stable training, gradient clipping was utilized, limiting the maximum gradient to within 1. In addition, data-augmentation strategies, including random cropping, horizontal flipping, and vertical flipping, were adopted to enhance the model’s robustness.

In the testing phase, the fold from the 5-fold cross validation that showed the best performance was used for testing to obtain the final performance metrics. Simultaneously, validation was conducted on other datasets to further assess the model’s robustness and generalization ability.

Overall, the experimental design was intended to evaluate the model’s performance comprehensively, effectively, and impartially while ensuring the reproducibility of the experimental results.

#### 4.2.4. Experiment Metric

In these experiments, mean average precision (mAP), precision, recall, and frames per second (fps) were selected as evaluation metrics.

1. mAP (mean average precision): mAP is a common measure for assessing the performance of object detection or instance segmentation tasks. It computes the average of the area under curve (AUC) of precision and recall of the predicted bounding boxes. mAP evaluates the performance of the model at all thresholds comprehensively, and its mathematical expression is
  (17) $$\mathrm{mAP}=\frac{1}{\left|Q\right|}\sum_{q\in Q}\frac{1}{m_{q}}\sum_{k=1}^{m_{q}}P_{k}^{q}$$
  where *Q* is the set of all queries, $m_{q}$ is the number of relevant documents for the *q*-th query, and $P_{k}^{q}$ is the precision of the *k*-th document. A higher mAP signifies better model performance.
2. Precision: Precision is a metric used to assess the accuracy of the model prediction, and its mathematical expression is
  (18) $$\mathrm{Precision}=\frac{TP}{TP+FP}$$
  where TP represents the number of true positives, and FP represents the number of false positives. In this task, precision reflects the proportion of correctly predicted disease regions out of all predicted regions.
3. Recall: Recall is a metric used to assess the coverage of the model prediction, and its mathematical expression is
  (19) $$\mathrm{Recall}=\frac{TP}{TP+FN}$$
  where FN represents the number of false negatives. In this task, recall reflects the proportion of correctly predicted disease regions out of all actual disease regions.
4. FPS (frames per second): FPS is a metric used to evaluate the computational efficiency of the model. In practical applications, especially in scenarios requiring real-time processing, FPS is critical. A higher FPS indicates that the model can process more images in a short period, denoting higher computational efficiency.

These four metrics jointly evaluate the performance of the model. mAP, precision, and recall reflect the prediction quality of the model from different perspectives, while fps indicates the computational efficiency of the model. In this task, the aim is to find a model that can detect tomato diseases with high precision and high computational efficiency.

## 5. Conclusions

With the rapid development of artificial intelligence and deep learning technology, their applications in the agricultural sector are becoming increasingly widespread, particularly in the domain of plant disease detection. In this context, this study focuses on the problem of high-precision detection of tomato diseases. As tomatoes are an essential fruit crop globally, their yield and quality are directly related to the economic benefits of agriculture and food safety issues. The goal of this work is to build a high-precision tomato disease detection system using deep learning technology, assisting agricultural workers in the timely and accurate identification of tomato diseases and thereby enabling effective preventive measures.

To accomplish this goal, a tomato disease image dataset was first constructed, and a NanoSegmenter model based on the Transformer structure was proposed. Lightweight techniques such as inverse bottleneck technology, quantization, and sparse attention mechanism were employed to optimize the model’s performance and computational efficiency. Experimental results demonstrated the outstanding performance of the model in tomato disease detection tasks with an accuracy of 0.98, recall of 0.97, and mIoU of 0.95. This implies that the model can accurately identify tomato diseases and successfully distinguish diseases from healthy tomatoes in most cases. Additionally, the model exhibited excellent computational efficiency, primarily attributable to the lightweight methods adopted. These methods effectively reduced the model’s parameter count and computational complexity, thereby enhancing the model’s inference speed, enabling it to reach up to 37 FPS.

Despite some positive outcomes, certain limitations in this research were recognized. Firstly, although the model performed well on the test set, its performance might be influenced by the distribution and quality of the dataset. Therefore, to improve the model’s generalizability, it is necessary to collect more data in future work, especially for those rare or complex disease types. Secondly, despite the model’s computational efficiency, it might still face challenges running in resource-limited environments, such as embedded or mobile devices. Thus, it is essential to further explore more effective model optimization and compression techniques.

Regarding future work, further exploration and improvement are planned from the following aspects: firstly, considering the use of semi-supervised or self-supervised learning methods to utilize unlabeled data, thereby enhancing the model’s generalization capability and robustness; secondly, trying to incorporate more advanced lightweight methods and neural network architectures into the model to further improve its performance and efficiency; finally, it is also worth studying the model’s operation on mobile or embedded devices to meet the needs of practical applications.

In summary, this study provides an effective solution for high-precision detection of tomato diseases by constructing a deep learning model. Furthermore, this work suggests some directions for improvement and expansion, offering insights and references for future research. 

## Figures and Tables

**Table 1:** Tomato disease detection accuracy experimental results.

Model	Precision	Recall	mIoU	FPS
NanoSegmenter	0.98	0.97	0.95	30
DeepLabv3+ [19]	0.97	0.96	0.93	27
DeepLabv3 [20]	0.96	0.95	0.91	26
UNet++ [21]	0.94	0.93	0.90	23
PSPNet [22]	0.93	0.91	0.88	21
SegNet [23]	0.91	0.90	0.86	20
UNet [24]	0.90	0.88	0.85	18
FCN [25]	0.88	0.86	0.83	15

**Table 2:** Experimental results on other dataset.

Model	Precision	Recall	mAP on Wheat Dataset
YOLOv5	0.61	0.58	0.59
SSD	0.58	0.53	0.55
NanoTransformer + Detection Network	0.61	0.57	0.58
			Accuracy on pear dataset
ResNet50	0.95	0.92	0.93
VGG19	0.91	0.89	0.91
AlexNet	0.88	0.87	0.89
NanoTransformer + Softmax	0.97	0.92	0.94

**Table 3:** Impacts of various lightweight techniques on model parameter quantity, computation, and GPU memory usage.

Method	Parameter Quantity	Computation	GPU Memory Usage
Inverted Bottleneck	Decrease by 30%	Decrease by 20%	Decrease by 20%
Sparse Attention	No Change	Decrease by 50%	Decrease by 30%
Quantization	No Change	Decrease by 90%	Decrease by 75%

**Table 4:** Experimental results of the impact of different lightweight methods on the performance of NanoSegmenter on various hardware platforms.

Method	Inverted Bottleneck	Quantization	Sparse Attention	Precision	Recall	mIoU	FPS1 ${}^{1}$	FPS2 ${}^{2}$	FPS3 ${}^{3}$
ours	None	None	None	0.98	0.97	0.96	14	11	27
ours	√	None	None	0.98	0.97	0.95	31	18	42
ours	None	√	None	0.97	0.96	0.94	32	18	44
ours	None	None	√	0.96	0.95	0.93	33	18	48
ours	√	√	None	0.95	0.94	0.92	34	19	50
ours	None	√	√	0.94	0.93	0.91	35	22	49
ours	√	None	√	0.93	0.92	0.90	36	19	51
ours	√	√	√	0.92	0.91	0.89	37	31	51
ShuffleNet	None	None	None	0.92	0.90	0.90	33	15	48
MobileNet	None	None	None	0.93	0.92	0.92	35	21	45

${}^{1}$ FPS on Jetson Nano. ${}^{2}$ FPS on Raspberry Pi. ${}^{3}$ FPS on smartphone.

**Table 5:** Category distribution and corresponding proportions within the total count in the dataset.

Category	Number of Images	Proportion
bacterial spot	468	0.030
early blight	1580	0.103
healthy	523	0.034
late blight	1478	0.096
leaf mold	2495	0.162
Septoria leaf spot	2510	0.163
spider mites: two-spotted spider mite	1569	0.102
target spot	2534	0.165
mosaic virus	1522	0.099
yellow leaf curl virus	504	0.033

**Table 6:** Category distribution within the dataset after data augmentation.

Category	Number of Images	Proportion
bacterial spot	2534	0.104
early blight	2534	0.104
healthy	2534	0.104
late blight	2534	0.104
leaf mold	2534	0.104
Septoria leaf spot	2534	0.104
spider mites two-spotted spider mite	2534	0.104
target spot	2534	0.104
mosaic virus	2534	0.104
yellow leaf curl virus	2534	0.104
Total	25,340	1.000
